# Hospital and economic burden of influenza-like illness and lower respiratory tract infection in adults ≥50 years-old

**DOI:** 10.1186/s12913-019-4412-7

**Published:** 2019-08-19

**Authors:** Cecilia Trucchi, Chiara Paganino, Andrea Orsi, Daniela Amicizia, Valentino Tisa, Maria Francesca Piazza, Domenico Gallo, Simona Simonetti, Bruno Buonopane, Giancarlo Icardi, Filippo Ansaldi

**Affiliations:** 1 Azienda Ligure Sanitaria (A.Li.Sa.), Genoa, Italy; 2 Department of Health Sciences, University of Genoa, Genoa, Italy; 3 Hygiene Unit, San Martino Polyclinic Hospital, Genoa, Italy; 4 Liguria Digitale S.p.A., Genoa, Italy

**Keywords:** Influenza-like illness, Lower respiratory tract infection, Comorbidities, Anti-influenza and pneumococcus immunization strategies, Syndromic surveillance system, Chronic condition data warehouse

## Abstract

**Background:**

Influenza-like illnesses (ILIs) and lower respiratory tract infections (LRTIs) cause substantial morbidity and mortality worldwide.

The study assessed the health and economic burden of ILI and LRTI according to age and comorbidities, since available evidence is limited and heterogeneous.

**Method:**

The prevalence of comorbidities, the seasonal incidence rates and the mean and per capita direct costs of ED accesses for ILI/LRTI, whether followed by hospitalization or not, recorded in adults aged ≥50 years over the last 6 years, in the referral hospitals located in the Genoese metropolitan area (Liguria, Italy) where the syndromic surveillance system is active, were evaluated through a retrospective observational study. Comorbidities were estimated through the Chronic Condition Data Warehouse that integrates multiple Medicare data sources. A comparison with the administrative healthcare International Classification of Diseases-9th revision-Clinical Modification (ICD-9-CM)-based data was also conducted.

**Results:**

The prevalence of subjects with ≥1 comorbidity ranged from 23.49 to 59.92%. The most prevalent all-age comorbidities were cardiovascular diseases and cancer. The overall ILI/LRTI incidence rate was 6.73/1000 person-years, almost double the value derived from routine data, and increased with age. The highest rates were observed in patients with renal failure and bronchopneumopathies. The mean cost of ED accesses/hospitalization for ILI/LRTI was €3353 and was almost twice as high in the ≥85 years as in the youngest age-group. The highest mean costs were observed in patients with renal failure and cancer. The per capita costs increased from €4 to €71 with age, and were highest in patients with renal failure and bronchopneumopathy.

**Conclusion:**

The burden of ILIs/LRTIs in terms of ED accesses and hospitalizations in adults aged ≥50 years is heavy, and is related to increasing age and, especially, to specific comorbidities.

These results could contribute to revising age- and risk-based anti-influenza and -pneumococcus immunization strategies.

**Electronic supplementary material:**

The online version of this article (10.1186/s12913-019-4412-7) contains supplementary material, which is available to authorized users.

## Background

Influenza-like illnesses (ILIs) and lower respiratory tract infections (LRTIs) cause substantial morbidity and mortality worldwide. Individuals at high risk, including the elderly, immunocompromised and chronically ill, suffer high rates of severe outcomes from viral and bacterial respiratory infections, and are therefore prioritized for vaccination. Nevertheless, few data on the impact of ILIs and LRTIs on health systems according to age, comorbidities and risk factors are available. These data are crucial to calibrating the health system response, developing age- and risk-based preventive strategies - i.e. recommendations for influenza and pneumococcus vaccination or antiviral therapy - guiding the proactive management of at-risk patients in primary care, implementing educational initiatives, and conducting future research.

Current Italian anti-influenza immunization strategies target subjects aged ≥65 years and those at risk of complications [1]. Furthermore, the 2017–2019 National Vaccine Prevention Plan recommends anti-pneumococcal vaccination for infants, the elderly (≥65 years) and subjects at risk of developing invasive pneumococcal diseases [2]. Nevertheless, during the last six influenza seasons, Italian and Ligurian influenza vaccine coverage (VC) rates have been suboptimal in the elderly (62.7–52% and 55.5–47.3%, respectively) and very low in at-risk subjects aged < 65 years (12–8.5% and 15.2–7.1%, respectively). By contrast, the incremental VC objectives for the recently introduced pneumococcal vaccination for the elderly are 55% in 2018 and 75% in 2019 [3].

Considering ILI and LRTI prevention, the implementation of age-based influenza vaccination in adults is one of the debated issues among decision-makers and stakeholders in several countries, in particular lowering (from 65 years to 50 years) the recommended age threshold for influenza vaccination is under evaluation.

Age is the main risk factor for the prevalent diseases [4] and 50 years is usually the transition age from a healthy state to first chronic condition and often reports on chronic diseases starts from this age [5].

In order to better design immunization strategy the availability of robust epidemiological data is fundamental.

Nevertheless, estimates of the burden of ILIs and LRTIs are impaired by the low accuracy, specificity and sensitivity of administrative healthcare data (AHD) based on the International Classification of Diseases (ICD), low sensitivity in capturing cases in which respiratory infections trigger severe diseases, and difficulties in detecting subjects with underlying medical conditions [6–8].To overcome these limits, we coupled data from the Chronic Condition Data Warehouse (CCDWH), recorded in the Liguria region since 2010, with those gathered from a chief complaint Syndromic Surveillance System (SSS) based on Emergency Department (ED) accesses. The ILIs/LRTIs SSS allows the alert state to be activated and the planned progressive response to ED crowding to be implemented during influenza seasons [9].

We assessed the health and economic burden of influenza and its complications in terms of annual incidence rates of ILIs and LRTIs requiring ED access, whether followed by hospitalization or not, and healthcare resource expenditures, according to age and comorbidities. A comparison with ILIs and LRTIs estimates derived from AHD, including ED and hospital discharge records (DRs), was also conducted in order to verify their accuracy.

## Methods

### Study context

The population of Liguria Region is the oldest one in Italy, therefore the chronic conditions are well represented in this area. Genoa is the regional capital of Liguria, an administrative region in Northern Italy. The Genoese metropolitan area (GMA), which contains the majority of the Ligurian population, is representative of the entire Ligurian region and no significant differences in terms of demographic structure and chronic diseases prevalence are found in Liguria. Further, in the era of ageing population, Liguria can be considered the reference of the other Italian regions and a predicting model of developed countries in particular for the evaluation of anti- influenza immunization strategy and of other age related vaccine preventable diseases.

### Study design

A descriptive retrospective study was conducted in order to assess the impact of ILIs and LRTIs in adults ≥50 years-old in terms of ED accesses, whether followed by hospitalization or not, recorded in the referral hospitals of the GMA, according to age and comorbidities, over the last six influenza epidemic seasons (November 1, 2011 - October 31, 2017). Since ILI and LRTI are acute infectious diseases, the healthcare pathway active in Italy begins in the most of cases with an ED access that is followed by hospitalization in a ward such as general medicine or respiratory disease unit depending on patient clinical condition. Therefore, we focused on the incidence of ED accesses and then we calculated the proportions of cases that required hospitalization.

### Administrative healthcare data

The AHD routinely collect data about ED accesses, hospitalizations, outpatients visits, diagnostic tests, treatments and medical fee exemptions financed by public health funding. They are transmitted by the Ligurian Local Health Units (LHUs) and hospitals to the regional health authority “Azienda Ligure Sanitaria” (A.Li.Sa), where the completeness and quality of data is verified and then sent to the national Ministry of Health. Healthcare data and costs routinely collected by the public funded LHUs and hospitals were used to assess the impact of ILIs and LRTIs in adults ≥50 years-old in terms of ED accesses, whether followed by hospitalization or not, and the chronic conditions linked to complications of ILI/LRTI recorded in patients resident in the GMA who attended the EDs.

### Syndromic surveillance system

The SSS monitors ILIs and LRTIs daily on the basis of ED accesses in the regional referral hospitals for adults, which serve an area with an adult population of 333,579 [10, 11]. Active since 2007, the SSS is coordinated by the Department of Health Sciences of the University of Genoa.

Syndrome coding, data collection, transmission and processing, statistical analysis to assess indicators of disease activity and alert thresholds, and signal response have been described by Ansaldi et al. [10, 11].

Briefly, an informatic system was developed in order to scan the chief complaint field for the word strings assigned to the single syndrome: the system provides for an automatic review of ED acceptance data folders, identifying suspected cases and subdividing them into the analysed clinical syndromes [(influenza-like illness (ILI), low-respiratory tract illness (LRTI)].

Based on these case definitions, each syndrome was identified by a combination of keywords that must appear in specific fields (anamnesis, case history and comments of the ED registration and triage software).

The case definitions of ILI and LRTI syndromes used during critical review of medical records, in addition to laboratory assays and imaging results, are described in Additional file 1. The confirmed cases are then entered in a specific database for each syndrome and contribute to the evaluation of the impact.

The results obtained through the SSS were compared with routine AHD based on ICD 9-CM codes (052.1, 112.4, 136.3, 480–487) suggestive of pneumonia or influenza, including ED and hospital DRs (primary and secondary diagnoses were considered). In Italy the national AHDs uses the 9th version of the ICD to classify diagnoses and procedures.

### Chronic condition data warehouse

The chronic conditions linked to complications of ILI/LRTI recorded in patients resident in the GMA covered by the SSS who attended the EDs were obtained through the Liguria CCDWH.

The CCDWH records data gathered from multiple Medicare data sources (hospital DRs, pharmaceutics, medical fee exemptions, outpatient visits and laboratory/imaging procedures) within a specified period by means of a predefined algorithm based on the codes assigned to specific diagnoses and procedures. Through a record-linkage system based on a civil registry database, residents’ histories of healthcare events are constructed in order to depict the chronic condition of each patient. All data are archived in a relational database (RDBMS) by means of big-data logic.

For the purpose of the study, only chronic conditions recognized as risk factors for complications of influenza by most developed countries, including Italy, were evaluated at the time of ED access (i.e. organ transplantation, renal failure, HIV infection, malignancies, diabetes, cardiovascular diseases, bronchopneumopathy, gastrointestinal diseases, neuropathy, autoimmune diseases, endocrine metabolic disorders and rare diseases) [1, 12]. The CCDHW include rare diseases such as infectious diseases, cancers, endocrine glands diseases, nutrition, metabolism and immune disorders, diseases of blood and hematopoietic organs, neurological diseases, cardiovascular diseases, gastrointestinal diseases, genito-urinary tract diseases, skin and subcutaneous tissue diseases, diseases of the osteomuscular system and connective tissue, congenital malformations and perinatal diseases. The concomitant presence of chronic conditions and ILI/LRTI was considered as “comorbidities”.

A unique code specific for each patient was used to link data from the SSS and the CCDWH and to evaluate the patient’s chronic conditions on the day of ED access.

To evaluate the presence of chronic conditions in residents of the area covered by the SSS, we searched the CCDWH platform and selected the period of each influenza season, the above-mentioned chronic conditions and the residential area of interest.

### Direct costs

Cost estimates of ED accesses, including admission to short observation, and the direct costs of all-cause hospitalizations were estimated from the perspective of the government as payer and they were obtained by scrutinizing the outpatient procedures performed in the ED and the Diagnosis-related group (DRG) system, respectively. In accordance with the regional reimbursement system, the evaluation of costs related to ED accesses followed by hospitalization included only the DRG-based costs. The costs were estimated yearly and stratified by risk factor and age group. The analysed direct costs represent the real values incurred by RHS and were not discounted, as this is a descriptive study that did not applied pharmacoeconomic modelling methods.

Furthermore, direct costs were adjusted for inflation (year 2018).

### Statistical analysis

ED accesses for ILIs/LRTIs were stratified by age-group (50–54 yrs., 55–59 yrs., 60–64 yrs., 65–69 yrs., 70–74 yrs., 75–79 yrs., 80–84 yrs. and ≥ 85 yrs. of age) and underlying comorbidities.

For each age-group, we calculated seasonal prevalence of each risk condition in inhabitants of the GMA at the beginning of the season. We considered each season as the period between November 1st and October 31st (following year). The median and range of the prevalence of seasonal comorbidities in each age-group in the overall observation period was then estimated.

The seasonal incidence rates (per 1000 person-years) of ED accesses for ILIs and LRTIs in GMA inhabitants, stratified by risk factor and age-group, were estimated considering as denominator the number of subjects residents in the catchment area of the above mentioned referral hospitals. The median and range of seasonal ILIs/LRTIs incidence in each age-group and risk condition in the overall observation period was then calculated.

Finally, the mean and per capita costs of ED accesses, including admission to short observation, whether or not followed by hospitalization, stratified by age-group and chronic condition, were estimated in the overall observation period.

## Results

The median and range of the seasonal prevalence of comorbidities in GMA residents aged ≥50 years are shown in Table 1. Although fairly high in all age-groups, the prevalence of subjects with at least one risk factor tended to increase with age, from 23.49% (21.63–24.79%) in 50–54-year-olds to 59.92% (51.43–64.09%) and 51.15% (42.24–57.15%) in the two oldest age-groups (80–84 and ≥ 85 years). The comorbidities showing the highest all-age prevalence were cardiovascular diseases (13.47% [10.14–15.97%]) and cancer (13.28% [11.84–14.87%]), followed by diabetes (9.83% [9.63–9.92%]), other endocrine-metabolic disorders (8.03% [7.11–8.39%]), bronchopneumopathy (7.58% [6.40–8.38%]) and neuropathies (6.08% [4.82–6.64%]). The prevalence of almost all the diseases considered increased markedly with age, reaching a peak in the elderly (25.85, 19.68, 16.93, 15.35, 11.50, 9.85% in patients with cardiovascular diseases, cancers, neuropathies, diabetes, bronchopneumopathies and other endrocrine-metabolic disorders, respectively).

**Table 1:** Prevalence (median, range) of comorbidities based on CCDWH in GMA residents, years 2011–2017

Risk factor	Age-group (years)								
	50–54	55–59	60–64	65–69	70–74	75–79	80–84	> 85	Total ≥ 50
Transplant	0.18 (0.17–0.18)	0.21 (0.18–0.26)	0.25 (0.20–0.32)	0.27 (0.14–0.35)	0.31 (0.21–0.38)	0.30 (0.16–0.44)	0.27 (0.08–0.46)	0.16 (0.07–0.24)	0.24 (0.16–0.32)
Renal failure	0.31 (0.29–0.38)	0.46 (0.41–0.53)	0.69 (0.58–0.84)	1.23 (0.97–1.40)	1.77 (1.48–2.19)	2.89 (2.25–3.25)	4.11 (3.30–5.03)	5.91 (4.32–7.23)	1.92 (1.17–2.32)
HIV/AIDS	0.78 (0.53–1.01)	0.50 (0.37–0.76)	0.36 (0.20–0.56)	0.23 (0.18–0.38)	0.23 (0.12–0.26)	0.10 (0.08–0.24)	0.06 (0.03–0.09)	0.02 (0.01–0.05)	0.32 (0.21–0.46)
Cancer	5.55 (4.97–6.23)	7.57 (7.24–8.23)	10.75 (9.73–11.23)	14.05 (13.38–15.18)	17.82 (15.68–19.43)	19.13 (16.76–22.75)	19.68 (17.11–22.75)	16.21 (13.18–20.5)	13.28 (11.84–14.87)
Diabetes	2.82 (2.61–2.97)	4.67 (4.54–4.80)	7.25 (6.71–7.45)	10.72 (10.07–11.06)	13.66 (12.76–14.11)	15.28 (14.94–15.38)	15.35 (14.60–16.75)	13.43 (12.26–14.77)	9.83 (9.63–9.92)
Cardiovascular Diseases	2.59 (2.00–2.88)	4.50 (3.79–5.11)	7.92 (6.46–8.46)	11.8 (9.74–13.57)	17.07 (12.67–19.16)	21.72 (16.46–25.31)	25.85 (19.37–32.25)	25.66 (17.14–33.87)	13.47 (10.14–15.97)
Bronchopneumopathy	4.54 (3.88–5.42)	4.92 (4.21–5.49)	5.66 (4.84–6.32)	7.19 (6.12–7.78)	8.84 (7.43–9.81)	10.09 (8.51–11.12)	11.50 (9.93–12.05)	10.58 (8.44–12.12)	7.58 (6.40–8.38)
Gastrointestinal diseases	2.38 (1.96–2.61)	2.23 (1.87–2.71)	2.07 (1.54–2.49)	2.07 (1.88–2.24)	2.25 (1.89–2.51)	2.19 (1.63–2.53)	1.83 (1.41–2.26)	1.36 (0.91–1.68)	2.08 (1.68–2.41)
Neuropathy	2.10 (1.59–2.33)	2.23 (1.87–2.64)	2.81 (2.22–3.20)	3.44 (3.01–3.67)	5.38 (4.46–5.57)	8.52 (6.83–9.18)	12.67 (10.37–13.52)	16.93 (12.81–18.65)	6.08 (4.82–6.64)
Autoimmune Diseases	2.59 (2.20–2.74)	2.88 (2.40–3.25)	3.08 (2.66–3.29)	2.95 (2.42–3.50)	2.45 (1.72–3.00)	1.85 (1.37–2.35)	1.41 (1.06–1.87)	1.04 (0.75–1.25)	2.37 (1.91–2.72)
Endocrine metabolic disorders	4.91 (4.81–5.11)	6.32 (6.03–6.48)	7.52 (7.13–7.86)	8.97 (8.32–9.29)	9.76 (8.33–10.31)	9.49 (8.19–10.67)	9.85 (7.61–10.93)	8.42 (6.23–10.48)	8.03 (7.11–8.39)
Rare Diseases	1.25 (1.03–1.44)	1.13 (0.97–1.29)	1.01 (0.95–1.2)	1.08 (0.90–1.14)	0.87 (0.66–1.09)	0.69 (0.47–0.78)	0.53 (0.46–0.57)	0.21 (0.11–0.39)	0.89 (0.74–1.04)
At least one risk factor	23.49 (21.63–24.79)	28.58 (26.83–30.12)	36.96 (35.95–37.27)	44.19 (40.76–48.43)	52.78 (46.26–57.23)	55.20 (51.49–59.56)	59.92 (51.43–64.09)	51.15 (42.24–57.15)	42.66 (38.54–44.77)
No risk factor	76.51 (75.21–78.37)	71.42 (69.88–73.17)	63.04 (63.01–64.05)	55.81 (51.57–59.24)	47.22 (42.77–53.74)	44.80 (40.44–48.51)	40.08 (35.91–48.57)	48.85 (42.85–57.76)	57.34 (55.23–61.46)

**Fig. 1:**
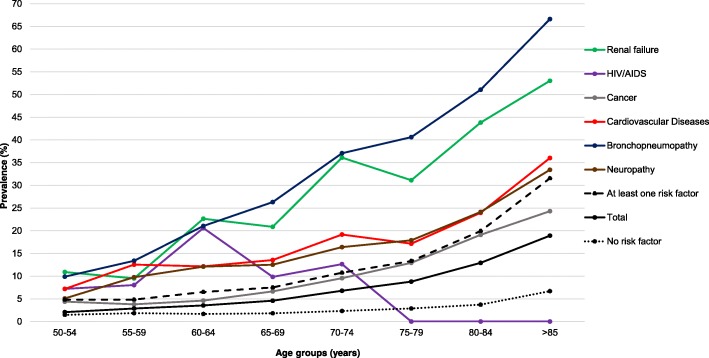
Incidence rate of ILI/LRTI ED accesses (/1000 residents), by comorbidity and age group, 2011–2017

Table 2 and Fig. 1, respectively, report the incidence of ED accesses for ILI/LRTI in the whole study period, stratified by age-group and comorbidity, and the incidence profiles of the most common comorbidities. The overall incidence rate in patients ≥50-year-old was 6.73 (range: 6.23–8.01) per 1000 person-years; the rate increased with age, from almost 2.08 in adults aged 50–54 years to 18.92 per 1000 person-years in the oldest elderly (≥85 years). The highest ILIs/LRTIs incidence rates in the study population were observed in patients with renal failure, bronchopneumopathies, followed by neuropathies, cardiovascular diseases and transplants: 38.32 (36.19–50.63), 37.41 (31.97–39.59), 21.43 (17.97–24.61), 20.63 (18.74–24.13) and 16.83 (4.87–25.89) per 1000 person-years, respectively. The incidence in patients with bronchopneumopathy and renal failure sharply increased from 9.88 to 66.63 and from 10.90 to 53.04 per 1000 person-years, respectively, from 50 to 54 years to ≥85 years (Fig. 1). Patients with gastrointestinal, cardiovascular and neurological diseases showed a less marked increase by age. Notably, the incidence of ILIs/LRTIs in subjects without risk factors was low and showed a less marked age-related increase than that observed in the highest risk categories.

**Table 2:** Incidence (median, range) of ILI/LRTI ED accesses (per 1000 inhabitants) based on the SSS and the CCDWH, years 2011–2017

Risk factor	Age-group (years)								
	50–54	55–59	60–64	65–69	70–74	75–79	80–84	> 85	Total ≥ 50
Transplant	12.20 (0–75.00)	2.33 (0–23.53)	0 (0–0)	16.72 (0–27.40)	16.15 (0–54.05)	26.59 (0–36.36)	20.94 (0–80.00)	0 (0–0)	16.83 (4.87–25.89)
Renal failure	10.90 (0–27.03)	9.51 (0–29.15)	22.66 (0–34.57)	20.87 (10.75–48.43)	36.11 (19.28–58.01)	31.13 (20.31–67.88)	43.83 (28.91–57.97)	53.04 (46.85–59.89)	38.32 (36.19–50.63)
HIV/AIDS	7.20 (4.45–23.87)	8.05 (0–20.51)	20.61 (0–28.14)	9.84 (0–36.36)	12.66 (0–52.63)	0 (0–51.28)	0 (0–111.11)	0 (0–0)	11.20 (8.22–13.95)
Cancer	4.40 (2.86–6.29)	3.81 (2.65–4.77)	4.63 (3.95–7.63)	6.65 (3.36–8.6)	9.56 (6.93–11.52)	12.93 (10.27–18.91)	19.11 (15.73–23.83)	24.31 (18.93–26.84)	12.11 (9.97–14.32)
Diabetes	3.92 (2.82–6.23)	6.84 (1.07–8.12)	7.90 (6.67–14.2)	7.03 (5.96–9.27)	15.15 (10.47–18.25)	17.04 (12.55–22.38)	20.13 (15.76–21.84)	26.08 (17.33–28.73)	14.92 (11.93–18.16)
Cardiovascular Diseases	7.18 (3.47–10.28)	12.55 (9.58–15.15)	12.16 (6.23–18.47)	13.55 (8.22–15.12)	19.16 (16.25–19.65)	17.15 (15.63–22.84)	23.97 (18.16–31.5)	36.03 (28.57–39)	20.63 (18.74–24.13)
Bronchopneumopathy	9.88 (6.79–12.38)	13.39 (6.80–15.62)	21.06 (15.94–24.81)	26.32 (18.06–33.31)	37.08 (28.52–42.81)	40.63 (36.35–54.18)	51.07 (35.77–59.36)	66.63 (58.91–70.78)	37.41 (31.97–39.59)
Gastrointestinal diseases	6.59 (1.64–11.95)	6.98 (2.16–12.9)	11.18 (4.66–14.97)	15.72 (10.94–22.08)	15.23 (4.78–20.98)	26.21 (14.81–30.14)	19.73 (13.25–24.32)	36.58 (6.80–66.67)	15.46 (13.77–16.53)
Neuropathy	5.10 (2.27–9.03)	9.77 (6.39–15.55)	12.09 (9.17–19.05)	12.52 (3.27–15.77)	16.40 (13.34–24.68)	17.90 (15.91–25.76)	24.15 (13.52–28.26)	33.43 (25.35–35.76)	21.43 (17.97–24.61)
Autoimmune Diseases	3.78 (1.82–7.56)	4.90 (3.36–9.23)	3.49 (0–8.79)	4.79 (0–7.84)	7.71 (2.04–11.2)	18.20 (13.79–26.75)	14.99 (5.54–30.00)	25.04 (16.67–44.82)	7.92 (7.02–9.69)
Endocrine metabolic disorders	2.35 (0.86–6.72)	3.43 (2.19–4.50)	5.47 (3.44–7.49)	6.14 (2.85–9.63)	8.97 (5.74–10.10)	12.57 (8.90–19.71)	17.17 (11.66–23.26)	22.64 (15.47–31.77)	9.18 (8.30–12.13)
Rare Diseases	3.17 (0–16.86)	3.97 (0–9.78)	0.99 (0–24.45)	4.34 (0–7.38)	6.43 (5.03–21.33)	10.19 (0–24.59)	14.31 (3.32–25.81)	25.32 (0–58.82)	6.44 (3.20–10.39)
At least one risk factor	4.83 (3.36–6.74)	4.83 (4.25–5.65)	6.52 (4.76–8.62)	7.51 (7.51–8.97)	10.76 (9.70–12.55)	13.34 (12.26–16.89)	19.94 (16.42–22.74)	31.56 (26.68–36.13)	12.59 (11.81–14.78)
No risk factor	1.48 (0.93–2.21)	1.86 (1.28–2.41)	1.68 (1.39–2.03)	1.83 (1.64–2.72)	2.34 (1.71–2.67)	2.88 (2.05–3.45)	3.73 (3.30–5.13)	6.70 (4.88–8.42)	2.49 (2.10–2.88)
Total	2.08 (1.75–3.15)	2.88 (2.32–3.08)	3.55 (2.72–4.06)	4.59 (3.90–5.38)	6.80 (6.14–7.81)	8.79 (8.34–11.34)	12.93 (10.37–15.98)	18.92 (18.01–22.80)	6.73 (6.23–8.01)

The risk of ED access for ILI/LRTI in subjects with at least one factor was significantly higher than in those without risk factors (OR = 5.109 [95%C.I.: 4.805–5.431], *p* < 0.0001).

The median incidence rates of ED accesses for ILIs and LRTIs in ≥50-year-old residents during the study period were 6.73 per 1000 person-years (range: 6.23–8.01) according to the SSS and 3.4 (range: 2.6–4) according to the AHD. The 2:1 ratio between the rates obtained through SSS and AHD data was observed in all age groups (2.1 vs 1.1, 2.9 vs 1.2, 3.6 vs 1.4, 4.6 vs 2.1, 6.8 vs 2.7, 8.8 vs 4.2, 12.9 vs 6.2, 18.9 vs 10.9 per 1000 person-years).

The proportions of ILIs/LRTIs cases requiring hospitalization were 83.6 and 78.9% according to the SSS and the AHD, respectively.

An overall mean direct costs value obtained in the entire study period is reported in Table 3 in order to show a more representative estimate of the healthcare resources absorbed for the assistance of patients who accessed to the ED for ILI/LRTI followed or not by hospitalization.

**Table 3:** Direct costs (mean, not adjusted for inflation) of ILI/LRTI ED accesses (and hospitalization) estimated through AHD, years 2011–2017

Risk factor	Age-group								
	50–54	55–59	60–64	65–69	70–74	75–79	80–84	> 85	total ≥ 50
Transplant	€ 3933	€ 2100	–	€ 3613	€ 2646	€ 4153	€ 4348	–	€ 3544
Renal failure	€ 10,965	€ 2955	€ 3620	€ 3726	€ 3726	€ 4323	€ 3901	€ 3727	€ 3907
HIV/AIDS	€ 2195	€ 2363	€ 3333	€ 3899	€ 6077	€ 3769	€ 2374	–	€ 3197
Cancer	€ 2931	€ 2893	€ 3670	€ 3883	€ 3783	€ 3784	€ 4407	€ 3857	€ 3903
Diabetes	€ 2702	€ 3345	€ 3620	€ 3839	€ 4046	€ 3690	€ 3809	€ 3512	€ 3716
Cardiovascular Diseases	€ 2252	€ 3279	€ 3673	€ 3909	€ 4076	€ 3906	€ 3977	€ 3654	€ 3819
Bronchopneumopathy	€ 1430	€ 2479	€ 3379	€ 4052	€ 3763	€ 3762	€ 4037	€ 3621	€ 3673
Gastrointestinal diseases	€ 2560	€ 2637	€ 2730	€ 3266	€ 3469	€ 3408	€ 3916	€ 3561	€ 3282
Neuropathy	€ 2878	€ 3889	€ 3316	€ 4028	€ 4049	€ 3579	€ 3686	€ 3993	€ 3823
Autoimmune Diseases	€ 2605	€ 2187	€ 1500	€ 2971	€ 2983	€ 4272	€ 2839	€ 4413	€ 3185
Endocrine metabolic disorders	€ 1830	€ 1953	€ 2092	€ 4058	€ 4079	€ 3827	€ 3696	€ 3716	€ 3571
Rare Diseases	€ 2975	€ 1881	€ 2515	€ 1150	€ 6457	€ 3826	€ 3767	€ 3870	€ 3587
At least one risk factor	€ 2158	€ 2577	€ 3134	€ 3578	€ 3697	€ 3673	€ 3802	€ 3642	€ 3562
No risk factor	€ 1416	€ 1600	€ 1977	€ 2452	€ 3200	€ 3105	€ 2991	€ 3345	€ 2551
Total	€ 1779	€ 2108	€ 2777	€ 3297	€ 3616	€ 3595	€ 3699	€ 3593	€ 3353

Estimates of the economic burden of ILIs/LRTIs in terms of ED accesses and hospitalizations (Table 3 and Additional file 2) showed a mean cost of €3353 (€3257 adjusted for inflation) for each access in the overall study population; the figure was almost twice as high in the older elderly (€3593 and € 3394 adjusted for inflation) than in adults aged 50–54 years (€1779, and € 1837 adjusted for inflation). The same ratio between the 50–54-year age group and the elderly was observed among subjects without risk factors (€1416 vs €3345 and € 1459 vs 3178 adjusted for inflation).

The mean costs (adjusted and not) of ILI/LRTI in patients with comorbidities ranged from about €3200 (autoimmune diseases and HIV/AIDS risk groups) to about €3900 (renal failure and cancer risk groups). In subjects without risk factors, the overall mean cost was €2551.

Finally, Table 4 and Additional file 3 report the per capita costs of ILI/LRTI in GMA residents, stratified by comorbidities and age group. On stratification by age group, the costs ranged from €4 in adults aged 50–54 years to €71 (€67 adjusted for inflation) in the oldest elderly. The per capita costs were highest in patients ≥50-year-old with renal failure and bronchopneumopathy: €166 and €136 (€154 and €130 adjusted for inflation) per capita, respectively.

**Table 4:** Per capita direct costs (not adjusted for inflation) of ILI/LRTI ED accesses (and hospitalization) estimated through AHD, years 2011–2017

Risk factor	Age-group								
	50–54	55–59	60–64	65–69	70–74	75–79	80–84	> 85	Total ≥ 50
Transplant	€ 78	€ 18	€ 0	€ 55	€ 52	€ 81	€ 146	€ 0	€ 52
Renal failure	€ 133	€ 40	€ 81	€ 97	€ 142	€ 181	€ 175	€ 211	€ 166
HIV/AIDS	€ 22	€ 21	€ 58	€ 53	€ 113	€ 38	€ 42	€ 0	€ 38
Cancer	€ 14	€ 11	€ 19	€ 26	€ 36	€ 54	€ 90	€ 97	€ 48
Diabetes	€ 13	€ 20	€ 32	€ 28	€ 58	€ 63	€ 75	€ 89	€ 55
Cardiovascular Diseases	€ 16	€ 43	€ 45	€ 50	€ 77	€ 74	€ 102	€ 137	€ 84
Bronchopneumopathy	€ 14	€ 33	€ 72	€ 107	€ 141	€ 169	€ 200	€ 247	€ 136
Gastrointestinal diseases	€ 17	€ 20	€ 30	€ 55	€ 56	€ 89	€ 79	€ 138	€ 52
Neuropathy	€ 16	€ 40	€ 44	€ 48	€ 71	€ 70	€ 87	€ 135	€ 83
Autoimmune Diseases	€ 12	€ 13	€ 6	€ 14	€ 21	€ 87	€ 57	€ 127	€ 27
Endocrine metabolic disorders	€ 6	€ 7	€ 11	€ 25	€ 36	€ 52	€ 66	€ 88	€ 36
Rare Diseases	€ 16	€ 8	€ 13	€ 4	€ 58	€ 41	€ 61	€ 88	€ 25
At least one risk factor	€ 10	€ 13	€ 20	€ 28	€ 40	€ 52	€ 74	€ 119	€ 47
No risk factor	€ 2	€ 3	€ 3	€ 5	€ 7	€ 9	€ 12	€ 22	€ 6
Total	€ 4	€ 6	€ 10	€ 15	€ 24	€ 33	€ 48	€ 71	€ 24

## Discussion

AHD-based evaluation of the real epidemiological and economic burden of ILIs/LRTIs and of the role of comorbidities in causing ED accesses and/or hospitalization displays weaknesses in terms of sensitivity and specificity, both in the capture of ILI/LRTI cases and in chronic disease estimates [13–18]. We aimed to overcome these limits by estimating ILIs/LRTIs through the SSS. This system displays high sensitivity in capturing suspected cases, as the chief complaints recorded by ED admission software are scanned for keywords suggestive of ILI/LRTI syndromes, and data folders are automatically reviewed. It also offers high specificity once each case captured has been critically reviewed according to case definitions.

Furthermore, we integrated SSS data with those from the recently implemented CCDWH, a useful tool to identify chronic conditions through specific inclusion and exclusion criteria and incorporating multiple data sources to guarantee the completeness of healthcare data. The SSS had previously proved effective, specific and sensitive in the surveillance of measles and respiratory tract infections [11, 19].

Our study supports previous evidence of the superiority of the SSS to the AHD in terms of performance. Indeed, the incidence rates of ED accesses for ILIs/LRTIs in adults aged ≥50 years, evaluated through the SSS, proved to be almost double those yielded by the AHD.

Indeed the performance of AHD in terms of specificity and sensitivity in capturing LRTI cases is suboptimal. This limit could be overcome using SSS that combines high sensitivity in identifying suspected cases obtained by scanning the chief complaint field for the word strings assigned to the single syndrome and automatic review of ED acceptance data folders and high specificity as a result of critical revision of each reported case according to the operative case definition [19]. An in-depth analysis of the performance of SSS compared to the AHD was previously published by Ansaldi F et al. in 2009 [20].

Furthermore, the high proportion of ILI/LRTI cases requiring hospitalization, as estimated by both the SSS and the AHD, demonstrates their severity and the appropriateness of ED accesses.

Notably, the incidence rates of ILIs/LRTIs obtained from the GMA AHD are substantially comparable to those reported in the literature, particularly in Western Europe. This is true especially when hospital DRs are evaluated, though available estimates of community acquired pneumonia (CAP) incidence in the elderly vary widely from 1.4 to 23.3 per 1000 inhabitants [17, 21–29]. This broad range of reported estimates may be mainly attributable to differences in study designs, case definitions, study populations, sampling procedures, and the performance of surveillance systems.

However, comparison among estimates of ED DRs is limited by the paucity of published data and differences in capturing systems (e.g. ICD-9 codes, number of diagnosis fields, sample representativeness) and in healthcare pathways in other countries [18, 30, 31]. In the USA, for instance, after short observation in the ED, the patient is either formally hospitalized or discharged, while in Italian hospitals ED observation may be extended up to 36 h before admission or discharge is ordered [32].

Regarding the prevalence of comorbidities in subjects attending the ED with ILIs/LRTIs, the CCDWH allows us to predict the main risk factors and their distribution by age group.

Our study confirmed previous evidence that several comorbidities (renal failure, bronchopneumopathy, neuropathy and cardiovascular diseases) increase the risk of ED access/hospitalization for ILIs/LRTIs, and that incidence rates vary according to the type of comorbidity: from 2.6 per 1000 in patients with rare diseases of various organs and systems to 38.3 per 1000 in those with renal failure. For instance, Curcio et al. reviewed the available published data from 2005 to 2013 on the prevalence of risk conditions for CAP and invasive pneumococcal diseases in the elderly. They found that diabetes, chronic heart disease and chronic obstructive pulmonary disease were the most frequently reported comorbidities (range: 7.6–28.5%, 6.9–25.8%, and 3.8–15.4%, respectively) [33]. A review by Mauskopf et al. estimated the probability of hospitalization by age and high-risk sub-group, and identified patients with cancer or immunosuppression (20.8%) and those with congestive heart failure or chronic lung diseases (20%) as the groups at highest risk of hospitalization in the event of influenza [34]. Furthermore, an analysis by Ewig et al. of the German nationwide quality performance data on hospitalized patients with CAP revealed that the most frequent comorbidities, according to the ICD-10-German Modification (GM), were cardiac comorbidity (19.2%), central nervous system disorders (13.8%), pulmonary comorbidity other than chronic obstructive pulmonary disease (COPD) (13%) and diabetes mellitus (11.9%) [35]. Notably, the distribution of comorbidities in patients attending the ED for ILI/LRTI by age-group showed an increasing trend. The greatest risk factors, i.e. renal failure, cancer, diabetes, cardiovascular diseases, bronchopneumopathy, gastrointestinal diseases, neurological diseases, autoimmune diseases, were accompanied by a growing incidence of ILI/LRTI as age increased, with a 5–6.7-fold increase from 50 to 54 to ≥85 years of age. Bronchopneumopathies and renal diseases reached incidence levels of ED accesses > 50 per 1000 person-years in the oldest age-group.

Of relevance to immunization strategies is our finding that 60–64-year-old patients with bronchopneumopathies, neuropathies, renal diseases, gastrointestinal diseases and cardiovascular diseases showed a > 2-fold higher incidence than patients in the first age group for which influenza immunization is recommended (overall incidence in 65–69-year-old: 4.6 per 1000 person-years). Similarly, an incidence > 9.2 per 1000 person-years was observed in 50–54-year-olds patients with bronchopneumopathy, and in 55–59-year-old patients with cardiovascular diseases, bronchopneumopathy and neuropathies. Thus, age alone seems to constitute a weak risk factor for ILI/LRTI-related ED access, since the estimated incidences are low in subjects without risk factors.

Few studies have coupled stratification by “comorbidity” with stratification by “age-group” to evaluate the burden of ILIs/LRTIs and, to our knowledge, no estimates are available in terms of incidence of ILIs/LRTIs stratified by the above-mentioned variables, since previous studies have reported the prevalence of each syndrome and comorbidity separately [36–39].

Thus, the CCDWH could constitute an important tool for estimating healthcare needs and improving the planning of healthcare activities, especially in aging populations, as in Western Europe. Moreover, since it integrates various regional AHD and considers a specific time range in identifying each comorbidity, it yields precise, updated estimates of the prevalence of the main chronic conditions among residents of the region. Indeed, data on the impact of co-morbidities are usually gathered from single AHD records, such as hospital DRs, which capture ICD codes in secondary diagnosis fields or procedures, or from active surveillance involving high resource expenditures [30, 36, 37, 39–42].

Finally, our evaluation highlighted the considerable economic impact of ILIs/LRTIs in terms of both ED access and hospitalization, particularly in the older elderly and in patients with renal failure, cancer, cardiovascular diseases and neuropathies. However, as we focused on direct medical costs estimated through the AHD, further evaluations should include direct non-medical costs and indirect costs.

Comparison between our economic results and those reported in the literature is limited by several factors. Firstly, available estimates evaluate hospitalization and ED accesses, ILI- and CAP-related costs separately. By contrast, our findings combine the costs of the complete healthcare pathway due to both ILI and LRTI. Furthermore, the rare evaluations of comorbidities have generally been performed on aggregated risk-groups based on severity. Finally, study designs, populations, periods, case definitions, healthcare settings, and reimbursement systems differ among studies and countries. Indeed, the mean cost of CAP hospitalization in elderly patients ranges between about € 4500 and € 8301 [15–17]. Regarding influenza-related hospitalization costs, a recent cross-sectional study involving elderly patients admitted to 20 Spanish hospitals during the 2013/2014 and 2014/2015 influenza seasons reported a mean cost per laboratory-confirmed influenza case of € 3.219 [43].

The strength of our evaluations is that they indicate costs per capita and per ED access/hospitalization, stratified by age-group and risk factor.

The main limits of our study are its small surveyed population, albeit comparable to those of other studies, and the lack of both immunization data among ILI/LRTI patients and etiologic ascertainment of ILI/LRTI syndromes [21, 24–28].

To address the problem of achieving the objectives of the current national anti-influenza immunization strategies, epidemiological and economic evaluations of the burden of ILIs/LRTIs are particularly important.

## Conclusions

The burden of ILIs/LRTIs in terms of ED accesses and hospitalizations in adults aged ≥50 years is heavy, and it is related to increasing age and, especially, to specific comorbidities. Although “at-risk” immunization strategies are highly resource-consuming and difficult to implement, they seem to be the most effective means of targeting patients with the highest risk of developing serious influenza complications, which impact both on the health of frail subjects and on healthcare costs. Our results could be used to populate epidemiological models to evaluate age- and risk-based immunization strategies and the effect of recommending anti-influenza and anti-pneumococcal immunization at an earlier age.

## Additional files

Additional file 1: Case definitions of ILI and LRTI syndromes. (DOCX 18 kb)

Additional file 2: Direct costs (mean) of ILI/LRTI ED accesses (and hospitalization) adjusted for inflation, years 2011–2017. (DOCX 21 kb)

Additional file 3: Per capita direct costs of ILI/LRTI ED accesses (and hospitalization) adjusted for inflation, years 2011–2017. (DOCX 20 kb)

## Data Availability

The de-identified datasets generated and/or analysed during the current study are not publicly available due to the data nature, but are available from the corresponding author on reasonable written request.

## Abbreviations

A.Li.Sa: Azienda Ligure Sanitaria
AHD: Administrative healthcare data
CAP: Community acquired pneumonia
CCDWH: Chronic Condition Data Warehouse
COPD: Chronic obstructive pulmonary disease
DR: Discharge record
DRG: Diagnosis-related group
ED: Emergency Department
GMA: Genoese metropolitan area
ICD: International Classification of Diseases
ILI: Influenza-like illness
LHU: Local Health Unit
LRTI: Lower respiratory tract infection
RDBMS: Relational database management system
SSS: Syndromic Surveillance System
VC: Vaccine coverage
