# Supplementary material for: Hospital and economic burden of influenza-like illness and lower respiratory tract infection in adults ≥50 years-old
Source: BMC Health Serv Res. 2019 Aug 19;19:585. doi: 10.1186/s12913-019-4412-7 (PMC6700791; doi:10.1186/s12913-019-4412-7)
Supplement: Supplementary file 1 — Case definitions of ILI and LRTI syndromes. (DOCX 18 kb) [file 12913_2019_4412_MOESM1_ESM.docx]

**Additional file 1. Case definitions of ILI and LRTI syndromes**

| **Syndrome** | **Case definition** |
| --- | --- |
| **ILI** | - statement by physician: patient with “suspected or confirmed Influenza” or “ILI”; - presence of fever and respiratory symptoms (sore throat OR cough OR phlogosis/inflammation of respiratory tract OR ...) recorded during the ED access; - symptoms reported by the patient within 7 days: fever and respiratory symptoms (sore throat OR cough OR phlogosis/inflammation of respiratory tract OR of ...); - presence of dyspnea or respiratory failure preceded by ILI OR fever and respiratory symptoms;   even if the access is due to potential non-infectious complications of influenza. |
| **LRTI** | - statement by physician: patient with “suspected or confirmed bronchitis, bronchiolitis, bronchopneumonia, bronchial pneumonia, ...” - presence of fever, respiratory symptoms and radiological finding of bronchitis, bronchiolitis, bronchopneumonia, bronchial pneumonia. |
