# Supplementary material for: Hospital and economic burden of influenza-like illness and lower respiratory tract infection in adults ≥50 years-old
Source: BMC Health Serv Res. 2019 Aug 19;19:585. doi: 10.1186/s12913-019-4412-7 (PMC6700791; doi:10.1186/s12913-019-4412-7)
Supplement: Supplementary file 2 — Direct costs (mean) of ILI/LRTI ED accesses (and hospitalization) adjusted for inflation, years 2011–2017. (DOCX 21 kb) [file 12913_2019_4412_MOESM2_ESM.docx]

**Additional file 2. Direct costs (mean) of ILI/LRTI ED accesses (and hospitalization) adjusted for inflation, years 2011-2017**

| **Risk factor** | **Age-group** | | | | | | | | |
| --- | --- | --- | --- | --- | --- | --- | --- | --- | --- |
|  | **50-54** | **55-59** | **60-64** | **65-69** | **70-74** | **75-79** | **80-84** | **>85** | **TOTAL ≥50** |
| **Transplant** | 4,117 € | 2,149 € | - | 3,702 € | 2,730 € | 4,278 € | 2,226 € | - | 3,279 € |
| **Renal failure** | 11,483 € | 3,024 € | 3,723 € | 3,746 € | 3,674 € | 3,855 € | 3,470 € | 3,428 € | 3,614 € |
| **HIV/AIDS** | 2,260 € | 2,418 € | 3,421 € | 4,085 € | 6,235 € | 3,860 € | 2,448 € | - | 3,291 € |
| **Cancer** | 3,061 € | 2,970 € | 3,805 € | 3,799 € | 3,731 € | 3,609 € | 4,148 € | 3,595 € | 3,744 € |
| **Diabetes** | 2,799 € | 3,435 € | 3,718 € | 3,780 € | 3,819 € | 3,520 € | 3,599 € | 3,304 € | 3,555 € |
| **Cardiovascular Diseases** | 2,326 € | 3,371 € | 3,770 € | 3,886 € | 3,934 € | 3,780 € | 3,807 € | 3,439 € | 3,677 € |
| **Bronchopneumopathy** | 1,477 € | 2,544 € | 3,476 € | 3,816 € | 3,656 € | 3,587 € | 3,864 € | 3,372 € | 3,523 € |
| **Gastrointestinal diseases** | 2,636 € | 2,716 € | 2,820 € | 3,087 € | 3,017 € | 3,259 € | 4,042 € | 3,399 € | 3,180 € |
| **Neuropathy** | 2,961 € | 4,007 € | 3,421 € | 3,774 € | 3,641 € | 3,328 € | 3,424 € | 3,649 € | 3,547 € |
| **Autoimmune Diseases** | 2,676 € | 2,260 € | 1,538 € | 2,892 € | 2,910 € | 4,157 € | 2,676 € | 3,812 € | 3,084 € |
| **Endocrine metabolic disorders** | 1,875 € | 2,014 € | 2,159 € | 3,981 € | 3,933 € | 3,622 € | 3,557 € | 3,442 € | 3,434 € |
| **Rare Diseases** | 3,051 € | 1,954 € | 2,663 € | 885 € | 5,892 € | 3,931 € | 3,884 € | 3,996 € | 3,546 € |
| **At least one risk factor** | 2,232 € | 2,652 € | 3,230 € | 3,480 € | 3,593 € | 3,543 € | 3,625 € | 3,436 € | 3,440 € |
| **No risk factor** | 1,459 € | 1,647 € | 2,037 € | 2,448 € | 3,287 € | 3,047 € | 2,821 € | 3,178 € | 2,534 € |
| **Total** | 1,837 € | 2,170 € | 2,861 € | 3,228 € | 3,545 € | 3,475 € | 3,522 € | 3,394 € | 3,257 € |
