# Supplementary material for: Hospital and economic burden of influenza-like illness and lower respiratory tract infection in adults ≥50 years-old
Source: BMC Health Serv Res. 2019 Aug 19;19:585. doi: 10.1186/s12913-019-4412-7 (PMC6700791; doi:10.1186/s12913-019-4412-7)
Supplement: Supplementary file 3 — Per capita direct costs of ILI/LRTI ED accesses (and hospitalization) adjusted for inflation, years 2011–2017. (DOCX 20 kb) [file 12913_2019_4412_MOESM3_ESM.docx]

**Additional file 3. Per capita direct costs of ILI/LRTI ED accesses (and hospitalization) adjusted for inflation, years 2011-2017**

| **Risk factor** | **Age-group** | | | | | | | | |
| --- | --- | --- | --- | --- | --- | --- | --- | --- | --- |
|  | **50-54** | **55-59** | **60-64** | **65-69** | **70-74** | **75-79** | **80-84** | **>85** | **Total ≥50** |
| **Transplant** | € 81 | € 18 | - | € 56 | € 54 | € 84 | € 75 | - | € 48 |
| **Renal failure** | € 139 | € 41 | € 83 | € 97 | € 140 | € 162 | € 156 | € 194 | € 154 |
| **HIV/AIDS** | € 23 | € 21 | € 59 | € 55 | € 116 | € 39 | € 43 | - | € 39 |
| **Cancer** | € 14 | € 11 | € 20 | € 25 | € 35 | € 51 | € 84 | € 90 | € 46 |
| **Diabetes** | € 14 | € 21 | € 33 | € 28 | € 55 | € 60 | € 71 | € 84 | € 52 |
| **Cardiovascular Diseases** | € 16 | € 44 | € 47 | € 49 | € 74 | € 71 | € 98 | € 129 | € 81 |
| **Bronchopneumopathy** | € 15 | € 34 | € 75 | € 100 | € 137 | € 161 | € 192 | € 230 | € 130 |
| **Gastrointestinal diseases** | € 18 | € 21 | € 31 | € 52 | € 49 | € 85 | € 81 | € 132 | € 50 |
| **Neuropathy** | € 17 | € 41 | € 45 | € 45 | € 64 | € 65 | € 81 | € 123 | € 77 |
| **Autoimmune Diseases** | € 12 | € 13 | € 6 | € 13 | € 20 | € 84 | € 53 | € 110 | € 26 |
| **Endocrine metabolic disorders** | € 6 | € 7 | € 12 | € 25 | € 34 | € 49 | € 63 | € 82 | € 35 |
| **Rare Diseases** | € 16 | € 9 | € 14 | € 3 | € 53 | € 42 | € 63 | € 90 | € 24 |
| **At least one risk factor** | € 11 | € 13 | € 21 | € 27 | € 39 | € 51 | € 70 | € 112 | € 45 |
| **No risk factor** | € 2 | € 3 | € 3 | € 5 | € 7 | € 8 | € 11 | € 21 | € 6 |
| **Total** | € 4 | € 6 | € 10 | € 15 | € 24 | € 32 | € 46 | € 67 | € 23 |
